# Micro- and Macroscopic Analysis of Fatigue Wear of Gear Wheel Top Layer—An Impact Analysis of Thermochemical Treatment

**DOI:** 10.3390/ma17133203

**Published:** 2024-07-01

**Authors:** Piotr Osiński, Włodzimierz Dudziński, Adam Deptuła, Rafał Łuszczyna, Marek Kalita

**Affiliations:** 1 Faculty of Mechanical Engineering, Wroclaw University of Science and Technology, 7/9 Łukasiewicza St., 50-370 Wrocław, Poland; piotr.osinski@pwr.edu.pl; 2 Faculty of Technical and Economic Sciences, Collegium Witelon—State University, 5A Sejmowa St., 59-220 Legnica, Poland; wlodzimierz.dudzinski@pwr.edu.pl; 3 Faculty of Production Engineering and Logistics, Opole University of Technology, 76 Prószkowska St., 45-758 Opole, Poland; a.deptula@po.edu.pl; 4 Faculty of Organization and Management—Department of Production Engineering, Silesian University of Technology, 26-28 Roosevelta St., 41-800 Zabrze, Poland; 5 KOMAG Institute of Mining Technology, Department of Machinery and Equipment, 37 Pszczyńska St., 44-101 Gliwice, Poland; mkalita@komag.eu

**Keywords:** analysis of surface, pitting, thermochemical treatment, residual austenite, gear pump

## Abstract

Today, there are many diagnostic methods and advanced measurement techniques enabling the correct diagnosis and assessment of the type and degree of wear of cogwheels (gears, pumps, etc.). The present study presents an analysis of the surface defects of a cogwheel of an oil pump prototype (3PW-BPF-24). The test object operated for a certain number of hours under controlled operating and environmental parameters. The damage to the surface layer was caused by fatigue phenomena and previous thermo-chemical treatment. On the basis of the significant percentage share (~30%) of residual austenite in the volume of the diffusion layer, a hypothetical conclusion was drawn about the suboptimal parameters of the thermo-chemical treatment process (in relation to the chemical composition of the analyzed pinion). A large number of research studies indicate that the significant presence of residual austenite causes a decrease in tooth surface hardness, the initiation of brittle cracks, a sharp decrease in fatigue strength, an increase in brittleness and a tendency to develop surface layer cracks during operation. High-resolution 3D scans of randomly selected pitting defects were used in the detailed study of the present work. It was indicated that the analysis of the morphology of surface defects allowed some degree of verification of the quality of the heat/chemical treatment. The martensitic transformation of residual austenite under controlled (optimum) repeated heat treatment conditions could significantly improve the durability of the pinion (cogwheel). In the case analyzed, the preferred treatment was the low-temperature treatment. The paper concludes with detailed conclusions based on the microscopic and macroscopic investigations carried out.

## 1. Introduction

Modern drive systems rely on precisely designed and manufactured gear wheels, which are crucial components for power transmission. The strength and durability of these elements are of paramount importance for the reliability of entire machines and devices. Pitting and micropitting, which are processes of fatigue wear on the surface of gear teeth, present significant engineering challenges, affecting the performance and longevity of gears. Advanced diagnostic techniques, such as scanning electron microscopy (SEM) and finite element analysis (FEA), allow for a detailed examination of these damage mechanisms. Furthermore, material properties and proper heat treatment play a key role in improving the durability of gear wheels. Monitoring the technical condition and diagnosing gears using modern methods, such as vibration analysis and thermography, enable the early detection of damage and planning of repair actions. The study also addresses issues related to the design and optimization of hydraulic systems, as well as the diagnostics and operation of hydraulic motors, which are integral parts of contemporary drive systems. To ensure clarity in the introduction of this article, the literature has been divided into the following thematic blocks: the mechanisms of gear teeth damage (pitting and micropitting), the condition monitoring and diagnostics of gear systems, material properties and heat treatment, and the diagnostics and testing of gear teeth. Additionally, the literature covers various supplementary topics: the design and optimization of hydraulic systems, the operation and diagnostics of hydraulic motors, metrology and surface analysis, wear phenomena and material analysis, and technical specifications and standards.

### 1.1. Review of Literature

#### 1.1.1. Mechanisms of Gear Teeth Damage (Pitting and Micropitting)

Pitting and micropitting are critical failure mechanisms that can significantly affect the performance and lifespan of gear systems. These phenomena involve the formation of small pits or craters on the surface of gear teeth, resulting from repeated stress and contact fatigue. The contact between gear teeth (gear wheels), colliding with each other, is considered one of the most complicated cases of contact interaction in the field of tribology [1]. The authors aimed to understand how and why pitting occurs in gear teeth, as well as its evolutionary mechanisms. For their research, they employed advanced analytical and experimental techniques to observe and analyze the processes leading to pitting formation. They utilized scanning electron microscopy (SEM), finite element analysis (FEA), and material fatigue tests to identify key factors influencing the initiation and development of pitting. The design process of gear wheels [2] must take into account the appropriate geometry of the teeth, the correct profile, dimensional tolerances, and material selection. The research was conducted using advanced experimental and analytical methods, including fatigue tests and scanning electron microscopy (SEM). Evaluating the influence of different lubrication conditions on micropitting was one of the main objectives. It was found that lubricants with higher viscosity and better protective properties effectively reduced the initiation and propagation of micropitting, while additives such as anti-wear and antioxidant agents improved the durability of gear teeth. In paper [3], researchers analyzed the failure mechanism of pitting in spur gear teeth using a semideterministic approach. Their aim was to understand these mechanisms by combining deterministic and probabilistic approaches. Through geometric modeling, stress analysis, and computer simulations, they identified that pitting initiation is strongly associated with stress concentrations at the tooth contact surfaces and surface microdefects. These studies provide valuable insights into pitting failure mechanisms, which may lead to the development of more durable and reliable gear teeth. Precision in manufacturing and the precise fitting of elements are crucial to ensure optimal system operation. Furthermore, recent research has shown the influence of surface roughness on the fatigue wear behavior of gear wheels. The proper lubrication of meshing gear wheels is crucial for reducing friction, wear, and failures [4]. In their investigation [5], researchers explored sandblasting’s impact on gear tooth micro-positioning and its evolution into macro-positioning. Sandblasting treatments were evaluated through twin-disc machine tests to mitigate micro-positioning in case-hardened gear teeth. The study aimed to understand sandblasting’s effect on micro-positioning in casehardened gear teeth and its transition to macro-positioning. Experimental gear tooth-to-tooth rig tests, including interrupted testing, monitored micro-positioning initiation and propagation on tooth flanks. Two cylindrical gear specimen variants were tested, one with surface hardening, typical for gears. Poletto et al. [6] introduced a novel method for objectively identifying gear damage through surface topography analysis. Using white light interferometry to measure 3D tooth surface topography, they introduced a new roughness parameter, surface motion orientation, facilitating an objective classification of gear damage types. Feng et al. [7] conducted a comprehensive review of vibration-based gear wear monitoring techniques. They addressed the limitations of current methods and provided a review to aid further development in the field. In their proposed methodology [8], researchers studied the interaction of gear wear and contact fatigue under elastohydrodynamic lubrication. Their modeling approach analyzed wear and fatigue using changes in oil film pressure and thickness. Another study [9] conducted an experimental and comparative analysis of gear tooth damage using a dynamic model. They explored detecting gear distortions based on rotation frequencies, crucial for effective monitoring. Lastly, Ref. [10] introduced a hybrid forecasting approach for predicting pitting growth on gear tooth surfaces. Their model, integrating empirical data and measured pitting surfaces, offered tailored predictions for specific gear pairs, enhancing durability forecasts.

Monitoring the technical condition and diagnosing the health of gear systems is crucial for ensuring their reliability, efficiency, and longevity. Accurate monitoring and diagnostic practices help detect early signs of wear, damage, or irregularities, allowing for preventive and predictive maintenance. Various advanced diagnostic methods, including vibration analysis, thermography, and spectrometry, are employed to monitor gear systems. These techniques enable the early detection of potential issues, reducing the risk of unexpected failures and extending the service life of the components.

#### 1.1.2. Condition Monitoring and Diagnostics of Gear Systems

Ensuring the longevity and efficiency of machinery often requires careful attention to various operational factors. One critical aspect is the regular inspection and timely replacement of worn out components to prevent unexpected failures. Additionally, selecting suitable materials and implementing effective cooling systems can significantly enhance performance under demanding conditions. Wang, Ismail, and Golnaraghi highlight the importance of these strategies in their study on gear damage monitoring through vibration analysis [11]. In paper [12], the authors outlined a method for assessing the energy and ecological efficiency of a multisource drive system, emphasizing the importance of selecting secondary energy source parameters. Using a multisource hydrostatic drive system as a case study, they analyzed energy consumption and ecological factors. In paper [13], the authors introduced a new hydraulic satellite pump/motor design and investigated its working mechanism failure. They highlighted low durability under specific pressure conditions with refined rapeseed oil, attributing the rapid wear of satellite teeth to bending and surface contact fatigue. In paper [14], the author discussed design optimization methods for hydraulic pumps to enhance efficiency and durability. They emphasized the significance of optimizing design parameters to minimize energy losses and improve gear pump reliability in diverse industrial settings.

Regular maintenance, an exchange of oil, and the monitoring of lubrication quality are important activities in maintaining appropriate reliability and endurance. In particular, lubrication plays an essential role in the case of using working liquids being oil-in-water emulsions [15] or the operation of pumps under thermal shock conditions [16]. State-of-the-art diagnostic methods such as the analysis of vibrations, thermography, or spectrometry enable one to monitor the condition of a gear wheel during its exploitation. They enable the detection of early signs of wear, damage, or irregularities, which renders it possible to undertake appropriate corrective measures before more serious failures occur. The literature contains many novel studies and suggestions concerning gear wheels and gear pumps. Tests of new materials, manufacturing technologies, the improvement of teeth geometry, and the optimization of lubrication aim to increase the endurance, efficiency, and reliability of these elements.

In work [17], the authors presented research results oriented toward testing the efficiency and reliability of different techniques for monitoring gear damage. Test cases with different kinds of teeth damage in the gear were used. As a result of the tests, it was found that beta kurtosis was a reliable diagnostic technique, and wavelet transform was useful for the visualization of inspection, particularly while using residue signals. In work [18], the authors concentrated on identifying future testing possibilities in the scope of damage diagnostics, using a quality analysis of the damage. In turn, in work [19], a simple monitoring method of the condition of gears operated in impulse environments was presented. The method of synchronous median turned out to be more resistant to the presence of impulse components of the signal, making it more reliable in estimating the state of individual components of the machine. The tests were based both on numerical and experimental data. Moreover, other publications present selected diagnostic methods concerning the assessment of gear wheel conditions and the detection of damage during the operation of a pump or a gear without any disassembly [20].

#### 1.1.3. Material Properties and Heat Treatment

Material properties and heat treatment play a vital role in determining the performance and durability of gear systems. The selection of appropriate materials and the application of optimal heat treatment processes are crucial for enhancing the strength, hardness, and wear resistance of gear teeth. Heat treatment techniques, such as thermochemical treatment, significantly impact the fatigue life and overall reliability of gear components. Research in this area focuses on understanding the effects of different heat treatment methods, material compositions, and surface modifications on the mechanical properties and longevity of gears. The literature in this section discusses various approaches to improving material properties and heat treatment processes to achieve a superior performance and extended service life of gear systems.

In addition to gear wheel diagnostics, it is worth noting that there are diagnostic methods for pump operation based on oil testing [21,22,23,24]. These methods utilize various techniques to analyze the condition of the lubricating oil within the pump system, providing valuable insights into the pump condition and performance. For instance, oil analysis techniques such as spectroscopy, chromatography, and viscosity measurement can detect contaminants, degradation products, and wear debris in the oil, indicating potential issues within the pump system [25,26]. Moreover, real-time monitoring systems incorporating sensors and algorithms enable the continuous assessment of the oil condition and pump operation, allowing for the early detection of abnormalities and predictive maintenance strategies [27]. These diagnostic methods play a crucial role in ensuring the reliability, efficiency, and longevity of pump systems in various industrial applications [28].

The damaged gear wheels are subjected to macroscopic and microscopic tests to obtain even better results [27]. The assessment methods of gear wheel wear after their disassembly, using microscopes such as focal differentiation microscopes made by Alicona, can deliver detailed information on the state of the surfaces of gear wheels and the degree of their wear.

#### 1.1.4. Diagnostics and Testing of Gear Teeth

The diagnostics and testing of gear teeth are essential for evaluating their performance, identifying potential issues, and ensuring the reliability of gear systems. Advanced analytical techniques and experimental methods are employed to assess the condition of gear teeth and detect any signs of wear, fatigue, or damage. These diagnostics include macroscopic and microscopic analyses, finite element analysis (FEA), and nondestructive testing (NDT) techniques. In paper [29], the authors presented an innovative approach to monitoring gear wear. They used a molding method and noncontact optical measurements, along with laser scanning confocal microscopy, to obtain detailed wear information without disassembling the gearbox. They analyzed surface roughness, wear severity, and wear depth during a lubricated endurance test. The methodology offers valuable insights for gear condition monitoring and can be applied to other conditions and geared transmissions. During a wear assessment of gear wheels with microscopes, specialized software, and a high resolution of images, the precise geometric measurement of external and internal dimensions of teeth, the profile of teeth, pitches, and tolerances [30] is carried out. Additionally, methods of finite element analysis (FEA) using software such as ANSYS enable a virtual analysis of the load and wear of gear teeth based on a numerical method. These methods are particularly useful for forecasting the behavior of gear wheels in different operational conditions, optimizing the design, and diagnosing problems related to strength and wear [31,32,33]. Macroscopic tests enable an assessment of gear pump condition, the identification of potential problem areas, and an evaluation of the remaining life and reliability of these devices. This process can incorporate an analysis of cracks, microcracks, changes in shape, and structural damage on the surfaces of teeth, pump frames, shafts, and other elements [34,35,36]. The authors of [37] reviewed the use of acoustic emission (AE) analysis for monitoring wind turbine gearboxes. They highlighted the challenges and opportunities in developing AE as an alternative to vibration analysis. Despite progress, standardized procedures are lacking, and existing models often overlook factors such as lubrication and surface roughness. Developing an analytical model to predict AE signatures based on specific gear parameters and faults is crucial for advancing AE. In [38], a method was developed to calculate time-varying mesh stiffness (TVMS) for gear teeth. A dynamic model of a planetary gear system with tooth damage was built, and simulations studied the impact on vibration responses. Experiments confirmed the model’s effectiveness, useful for diagnosing planetary gear system faults. In [39], transmission error (TE) was used to detect multiple cracks in gears. TE effectively identified the location and severity of cracks, proving more reliable than traditional vibration-based techniques.

#### 1.1.5. Hydraulic Systems: Design, Operation, Diagnostics, and Standards

In this area, various literature from the fields of hydraulic system design and optimization, hydraulic motor operation and diagnostics, metrology and surface analysis, wear phenomena and material analysis, and technical specifications and standards can also be referenced. These research areas aim to improve the efficiency, performance, and reliability of hydraulic systems by examining methods to enhance design parameters, monitor operational characteristics, evaluate surface quality, understand wear mechanisms, and adhere to industry norms and standards. Based on macroscopic observations, it is also possible to elaborate a strategy for the maintenance and repair of gear pumps to minimize the risk of failure and extend their life. Examples of work concerning macroscopic tests of fatigue wear in gear pumps include [26,27,28]. In work [40], experimental tests of macroscopic fatigue wear in gear pumps, concentrating on an analysis of failure and prevention, were conducted. In work [41], the authors carried out a macroscopic observation and analysis of fatigue damage in gear pumps under different operational conditions.

In turn, in work [42], the test concentrated on the macroscopic characteristics of gear pump failures under high-speed and heavy-load conditions. In these tests, an essential aspect includes the analysis of so-called pitting, i.e., the generation of losses on the tooth surfaces of gear wheels. Pitting is one of the main mechanisms of fatigue damage in gear pumps, which is why this phenomenon has key significance in macroscopic tests concerning the assessment of fatigue wear. The basic phenomenon of the top-layer wear of gear wheels operated in mediums such as hydraulic oil mainly includes pitting. It is a kind of damage that occurs on the surfaces of teeth and is characterized by the generation of micro- and macrocracks and electric discharges as a result of high contact stresses. Pitting can lead to a gradual deterioration of tooth surface quality, causing the erosion and flaking of material. This phenomenon is particularly dangerous because it can lead to serious damage to gear wheels and consequently to the failure of the whole machine. The tests performed concentrate on identifying, measuring, and describing pitting to better analyze its impact on the output and life of gear pumps.

Moreover, the operational properties of machine elements such as gear wheels mainly depend on the application of appropriate manufacturing technologies. In particular, due to the life of steel elements subjected to treatment, heat treatment giving optimum useful properties [43,44] has essential significance. A key issue includes the presence of residual austenite in the top layer of gear wheels subjected to heat treatment [45]. A significant number of research projects indicate that the presence of residual austenite, beyond a suitable limit value, reduces fatigue strength, hardness, and abrasion resistance [46,47,48].

## 2. Characteristics of Test Object

The analysis results of the form and size of the fatigue wear of the driven wheel of the prototype and the high-pressure gear pump (type: 3PW-BPF-24) are presented in this elaboration. The pump was subjected to an endurance test (the so-called labor resource test) [48]. The endurance test is a part of a compulsory set of tests to be performed before introducing the pump to the series production. The test, according to the accepted standard, requires a million cycles of loading. The period of one cycle is 2 s, during which the pump is loaded with the rated pressure at the half-cycle, and afterwards, it is unloaded. The pump rotational speed (according to the project assumptions) is determined in the scope from 600 to 6000 rev/min. However, the rotational speed, at which the pump was tested, complied with the rated speed of 1500 rev/min. The pump operational medium was hydraulic oil HL68, having wide application in medium-loaded hydraulic drive and control systems. According to the producer’s catalogue card [49] the HL68 oil guarantees a good protection of surfaces of lubricated elements (density: 0.885 g/cm^3^ at 15 °C and kinematic viscosity: 68 mm^2^/s at 40 °C). During the test, the oil temperature was maintained at the level of the recommended rated value, reaching 50 °C. However, the maximum temperature limit of operation for this unit is 80 °C. The object of the tests was operated 24 h, day and night, under controlled rated operational and environmental parameters (i.e., an appropriate rotational speed and a constant temperature) until one million cycles of loading were reached (about 24 days). After the endurance test, the hydraulic characteristics for the selected rotational speeds, n = 800, 1000, 1500, and 2000 rev/min, were determined. The condition, enabling us to approve of the pump design, according to the standard, includes a reduction in efficiency at any working pressure by a value not exceeding 8% in relation to the characteristics obtained directly after a factory run-in. After the completion of the labor resource test, the experimental unit was disassembled and each of its components was subjected to a detailed visual inspection and an analysis oriented towards the determination of the wear degree.

**Figure 1:**
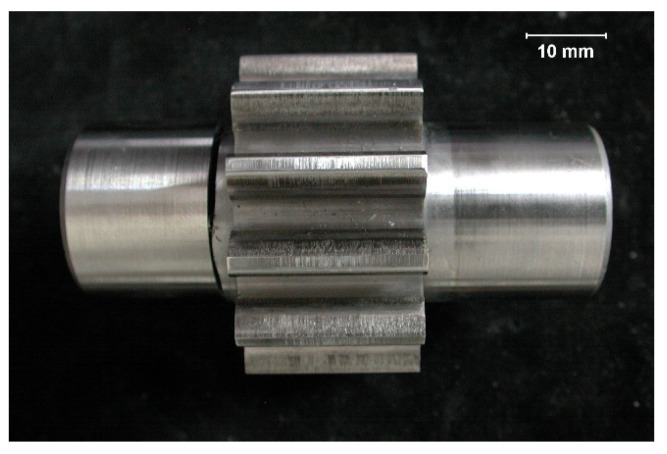
General view of gear wheel [27].

A general view of the analyzed pinion after the completion of the labor resource test is presented in Figure 1.

**Figure 2:**
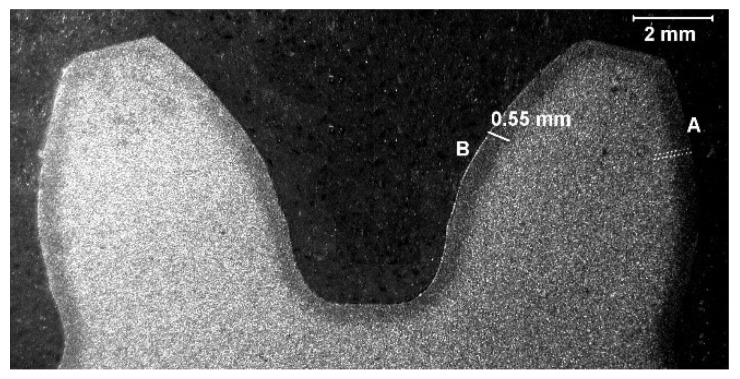
Tooth cross-section (visible changes in macrostructure resulting from the applied thermochemical treatment) [27].

The project assumptions required the manufacture of the pinion, subjected to tests, made of steel of the 18CrNiMo7-6 grade for carburization (acc. to the out-of-date standard PN-EN 10084:2002) or 17NiCrMo6-4 (acc. to PN-EN ISO 683-2:2022-07) and the attainment of the following properties due to applying thermochemical treatment:

- The thickness of the carburized layer (after mechanical working): 0.5–0.9 mm (marked in Figure 2);
- The core hardness: 29–42 HRC;
- The hardness of the top layer of working surfaces: 60–64 HRC.

The values given above were not fully reached due to high contents of residual austenite (which is described in a further part of this elaboration).

## 3. Macroscopic Tests Results

Macroscopic tests of gear wheel external surfaces were made via the naked eye and with the use of an SMT-800 stereoscopic microscope and a scanning microscope (Hitachi S3400N) [27].

**Figure 3:**
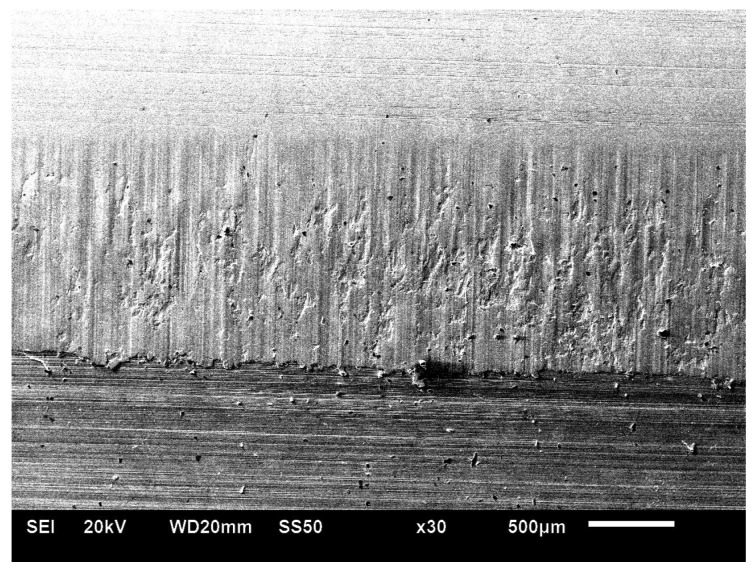
Macroscopic view of the tooth surface from the A side (Hitachi S3400N scanning microscope) [27].

**Figure 4:**
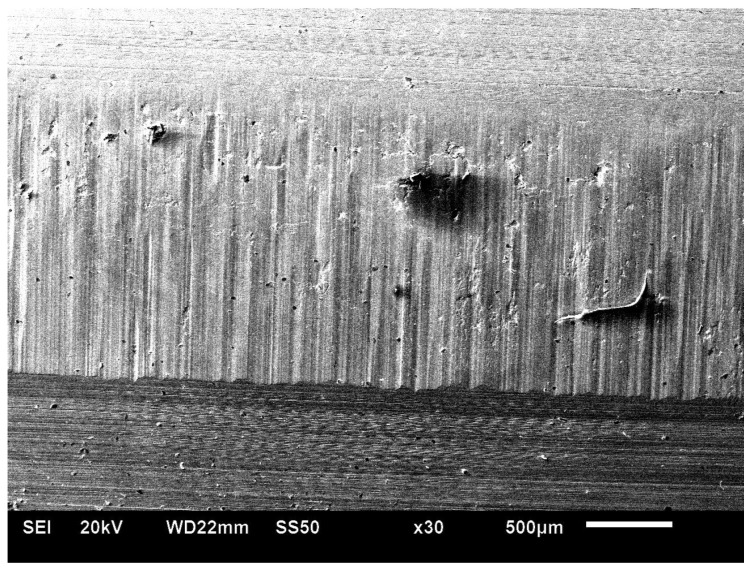
Macroscopic view of the tooth surface from the B side (scanning microscope Hitachi S3400N) [27].

On both sides of the external surface of teeth, traces of exploitation in the form of chippings and of working surface wear—Figure 3 and Figure 4 [27]—were seen.

After cutting off a specimen perpendicularly to the tooth, generating a line, and after regrinding and pickling it with use of the reagent Mi11Fe acc. to PN-H-04503:1961, some changes in the macrostructure resulting from the application of the surface thermochemical treatment to a depth of about 0.55 mm, as shown in Figure 2 [27], were noticed. Additionally, the macroscopic analysis of the external surfaces of the gear wheel also showed the presence of micropitting, which is a minute kind of superficial damage. Micropitting is caused by contact stress and cyclic loading, which leads to the generation of microdamage on the surface of teeth. The occurrence of micropitting may indicate the inappropriate operation of the constructional parameters of the gear wheel.

## 4. Microscopic and Hardness Test Results

Specimens for microscopic tests were cut off perpendicularly to the tooth, generating a line. Microscopic tests were conducted at magnification in the scope from 100× to 1000× in the non-pickling condition and after pickling with the reagent Mi1Fe according to the out-of-date standard PN-H-04503:1961. The metallographic microscope Epiphot 200, coupled with the digital film camera CCD Nikon, was used. The photographic documentation (of characteristic structures), related to the scope of work [27], is only and exclusively presented in the elaboration.

In the non-pickling condition, the occurrence of a small amount of non-metallic inclusions, mainly in the form of oxides spaced pointwise, was noticed. In the close-to-the-surface layer, no internal oxidation zone was spotted [27].

After pickling with the reagent Mi1Fe, a structure typical for carbonized, heat-treated objects was seen. The determination of the conventional depth of hardening after carbonization was carried out by a measurement of hardness at the cross-section along the normal surface to the carbonized surface, in the direction from the surface to the inside of the material, according to the standard PN-EN ISO 18203:2022-09. The measurements were taken with use of the Vickers method in the conditions according to PN-EN ISO 6507-1:2018-05. The Zwick 321 hardness tester, with a load of 1 kG (9.807 N) and acting within the time of 15 s, was applied. The average core hardness from five measurements, taken with use of the Vickers method, is 445 ± 3.25HV1 [27]. Table 1 and Table 2 present the results and visualize the hardness analysis from both sides of the tooth (A and B).

**Figure 5:**
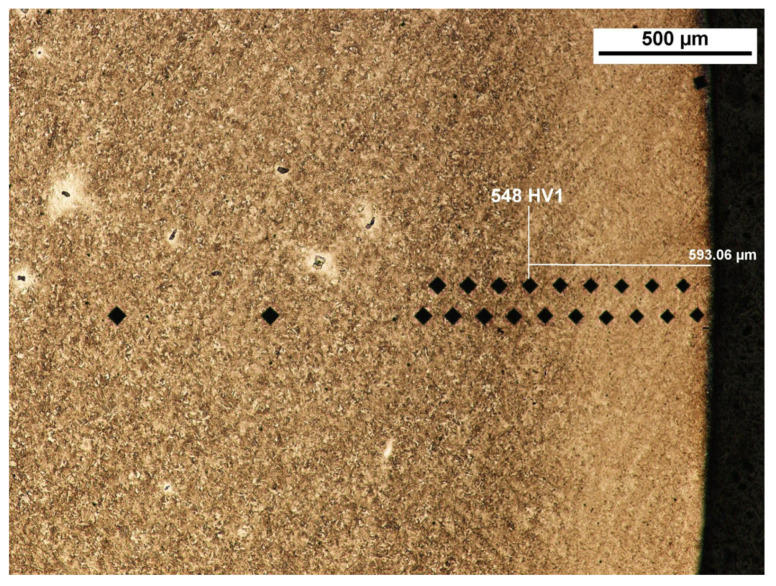
View of carbonized and hardened layer on the specimen’s cross-section from the A side with hardness distribution measurement markers (light microscopy, Mi1Fe pickling) [27].

**Figure 6:**
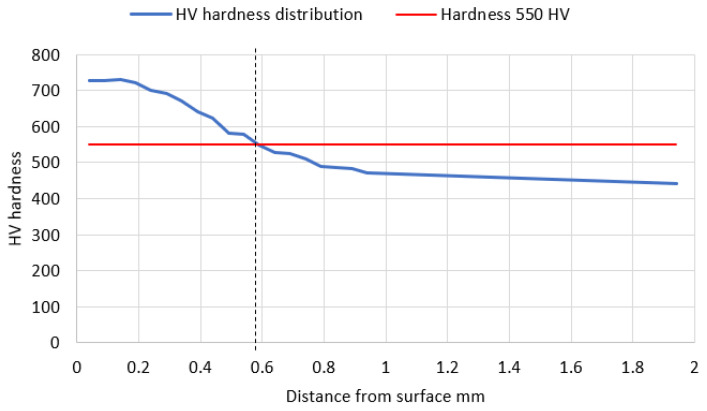
Hardness distribution along the normal to the carburized surface—from the A side (in the direction from the tooth surface to the material). Hardness 550 HV occurs at the depth of approximately 0.58 mm.

**Figure 7:**
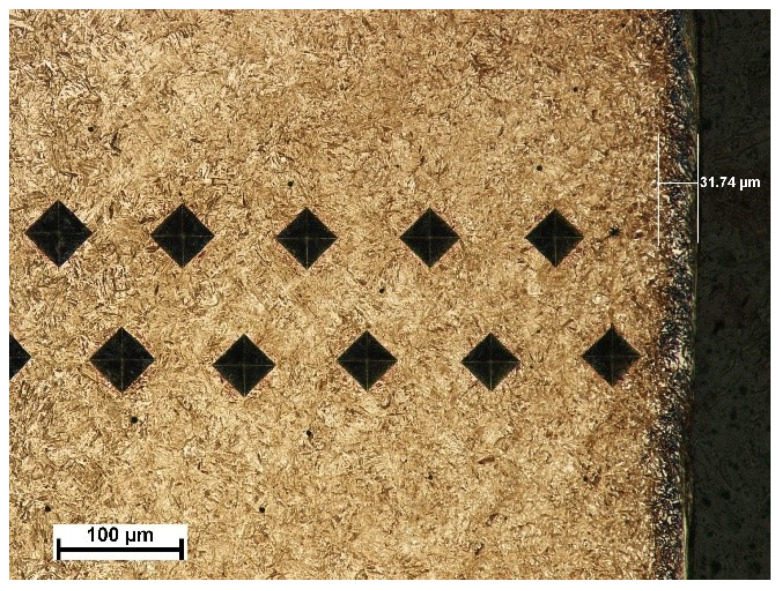
View of carbonized and hardened layer on the specimen’s cross-section from the A side with indentations of hardness distribution measurements and the layer of residual austenite (light microscopy, Mi1Fe pickling) [27].

**Figure 8:**
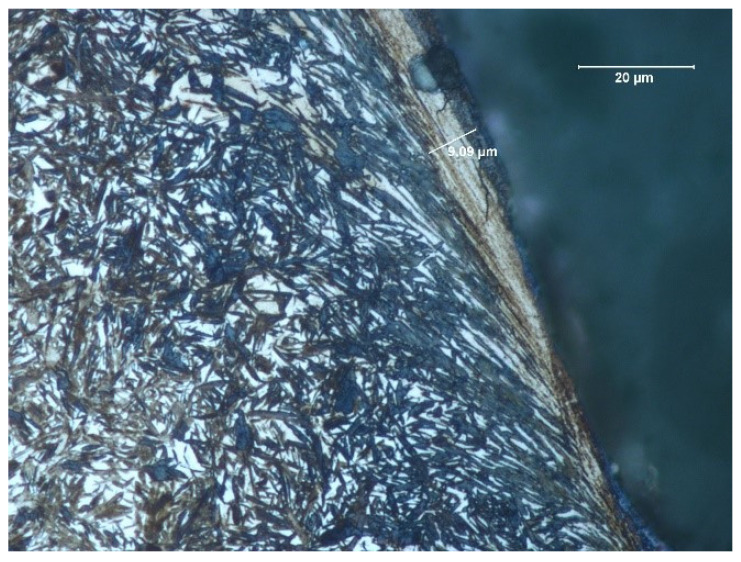
View of carbonized and hardened layer on the cross-section of the specimen from the A side—martensite with residual austenite with strongly deformed and cracked layer with a thickness of about 9 μm (light microscopy, Mi1Fe pickling) [27].

The depth of the tooth carbonized zone from side A is about 593 μm—Figure 5. The course of the hardening curve is shown in Figure 6. The occurrence of about 30% residual austenite, as well as the low-temperature tempered, fine acicular martensite of gradually decreasing carbon contents, was detected—Figure 7. A cracked, strongly deformed layer with a thickness of about 9 μm was seen on the tooth surface—Figure 8 [27].

**Figure 9:**
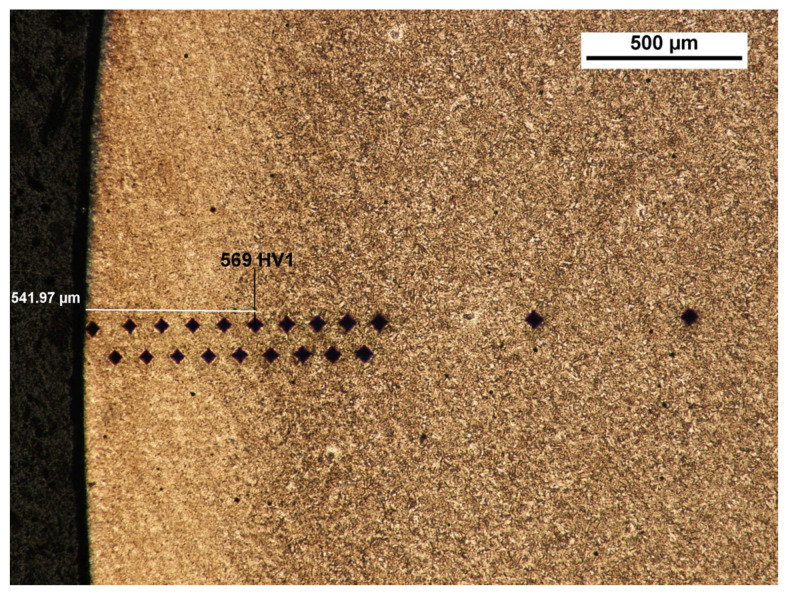
View of carbonized and hardened layer on the specimen’s cross-section from the B side with hardness distribution measurement markers (light microscopy, Mi1Fe pickling) [27].

**Figure 10:**
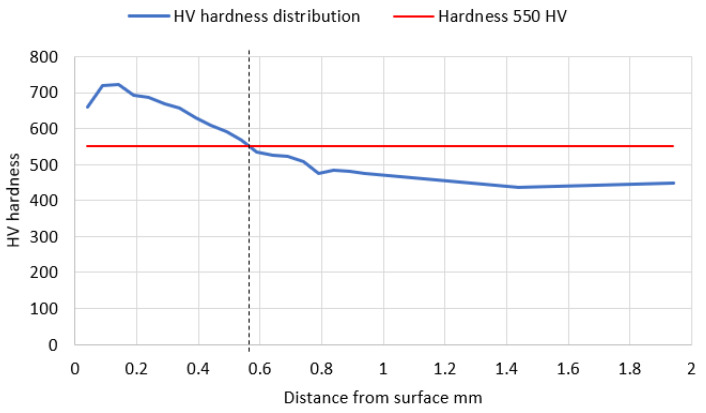
Hardness distribution along the normal to the carburized surface—from the B side (in the direction from the tooth surface to the material). Hardness 550 HV occurs at the depth of approximately 0.56 mm.

**Figure 11:**
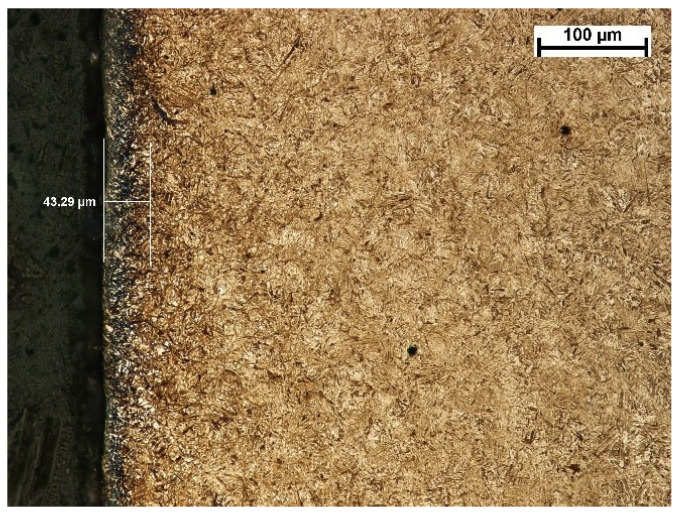
View of carbonized and hardened layer on the specimen’s cross-section from the B side with indentions of the hardness distribution measurements and the layer of residual austenite (light microscopy, Mi1Fe pickling) [27].

**Figure 12:**
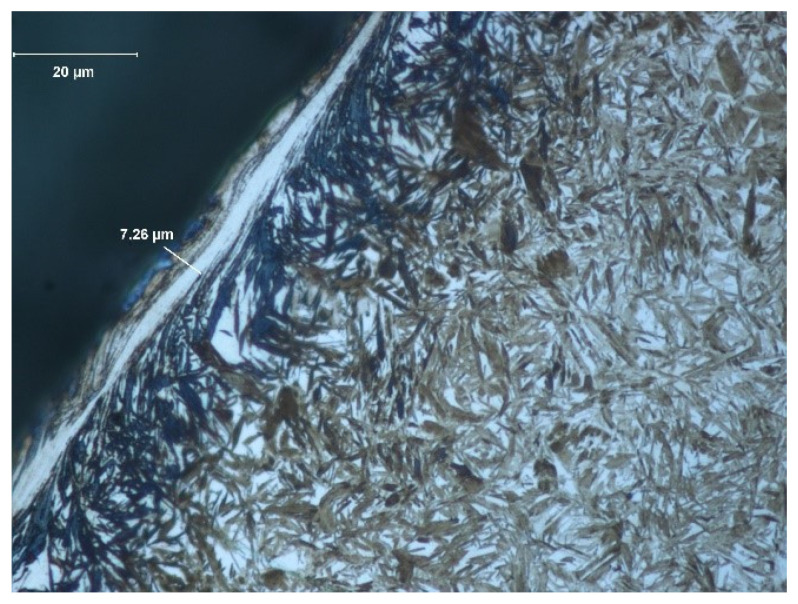
View of carbonized and hardened layer on the specimen’s cross-section from the B side—martensite with residual austenite with strongly deformed and cracked layer with a thickness about of 7.3 μm (light microscopy, Mi1Fe pickling) [27].

The depth of the tooth carbonized zone from the B side is about 542 μm—Figure 9. The course of the hardening curve is shown in Figure 10. In the close-to-the-surface zone from the B side at a depth of about 43.29 μm, the occurrence of about 30% residual austenite, as well as the low-temperature tempered, fine acicular martensite of gradually decreasing carbon contents, was seen—Figure 11. A cracked, strongly deformed layer with a thickness of about 7 μm was detected on the tooth’s surface—Figure 12 [27].

The structure of low-carbon martensite after heat treatment, typical for steels used for carbonization, was seen in the specimen core.

## 5. Analysis of the Material Chemical Composition

An analysis of the chemical composition of the pinion material was performed with the use of the spectral method, applying the PMI Master Pro spectrometer made by the Oxford Instruments company. The results of the chemical composition of the specimen material are presented in Table 3. The obtained values were listed and compared with the chemical composition of the 18CrNiMo7-6 steel, acc. to the out-of-date standard PN-EN 10084:2002.

In the conducted analysis of the chemical composition, with use of the spectral method, it can be stated that in the scope of the analyzed elements, the material of the specimen, subjected to tests, suits best steel that is of the grade 18CrNiMo7-6 for carbonizing, acc. to the out-of-date standard PN-EN 10084:2002. However, it cannot be classified into this grade due to the exceeded carbon content and lowered chromium content. Additionally, the specified composition does not have a direct equivalent in the group of alloy machine steels for carbonizing acc. to the standard PN-EN ISO 683-3:2022-07. The exact determination of the steel grade subjected to tests, acc. to the standards, compulsory at present, requires an additional series of strength tests [27]. Additionally, the results of the chemical composition analysis indicate the possible presence of other elements, which can have an impact on the material’s properties. Such tests enable the assessment of strength, hardness, and other essential mechanical parameters which are of key significance for the correct identification of the material and its useful properties.

## 6. Methodology of Test—Analysis of form and Size of Fatigue Wear of the Top Layer of Oil Gear Pump Wheel

**Figure 13:**
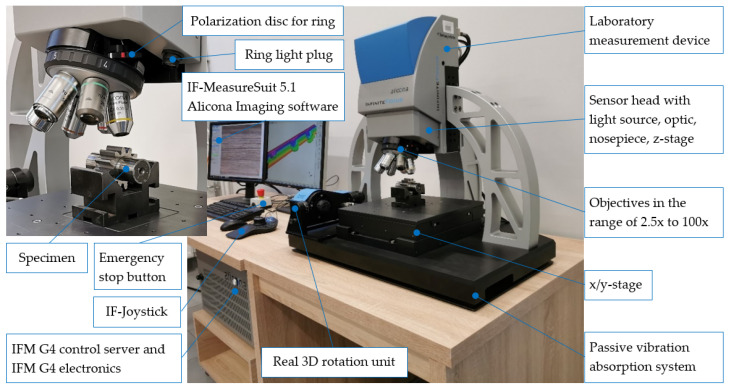
Characteristics of the measurement rig (Alicona InfiniteFocus G4 focal differentiation microscope).

The Alicona InfiniteFocus G4 focal differentiation microscope, which is a universal, precise and quick optical device used for the measurement of the profile geometry, roughness, and topography of a 3D surface, was used for an assessment of the kind and size of the top-layer wear—Figure 13.

Scans of individual areas of pitting breaches were realized (in relation to the need) with objectives in the 5× or 10× magnification. During digitalizing, a polarization diaphragm, which is in particular dedicated to strongly reflexive specimen, was used (it was a necessity in the case of applying the Focus-Variation technology). An analysis of the 3D surface topography was carried out according to the recommendations of the PN-EN ISO 25178-600:2019-05 standard.

Additionally, during the microscopic analysis, conducted with the use of the focal differentiation microscope Alicona InfiniteFocus G4, advanced algorithms of image processing, enabling the identification and measurement of the micropitting depth, were applied. Due to this, it was possible to obtain detailed information concerning the depth and dimensions of these defects, which was essential for the assessment of their impact on the strength and functionality of a gear wheel.

### Wear Assessment of Top-Layer Working Surfaces of the Pinion

**Figure 14:**
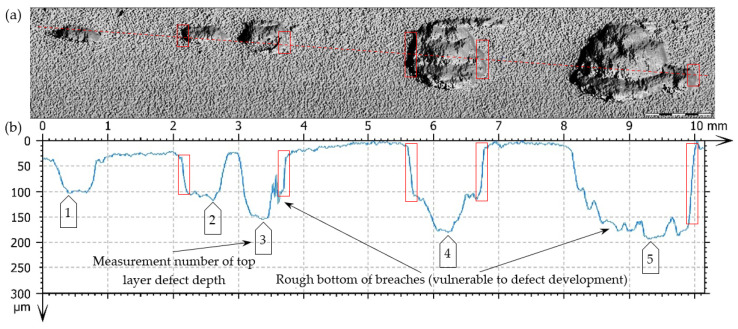
Fatigue pits (pitting) on the tooth’s working surface—magnification 5×. (**a**) Photo simulation, (**b**) profile curve, -----—line of cross-section, □—indication of breach edges inclined to the surfaces, subjected to analysis at an angle close to 90° on the cross-section.

As a result of taking superficial 3D scans, superficial fatigue damage (characterized by breaches of oval shapes and sharp edges, inclined perpendicularly to the surface of the teeth under analysis)—Figure 14—was diagnosed.

**Figure 15:**
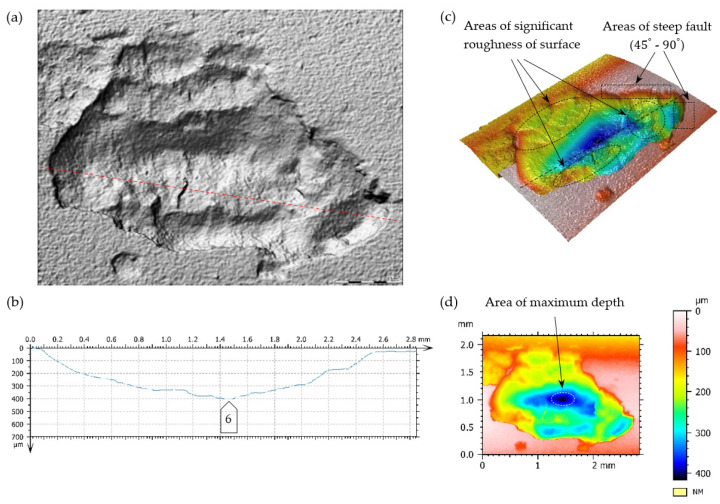
Fatigue pit (pitting) on the tooth’s working surface—magnitude 5×. (**a**) Photo simulation, (**b**) profile curve, (**c**,**d**) 3D superficial view—pseudocolour of surface with the depth visualization, -----, -----—lines of cross-section.

The observed breaches, generated as a result of the material chipping, have different dimensions and a rough bottom, and they occur locally or on a much smaller scale on a bigger tooth surface (micropitting)—in particular below the pitch diameter of the pinion. A linear arrangement of the described defects was observed in some places—Figure 15.

The advancing chipping of the material particles, which is presented in Figure 15, can lead, in extreme conditions, to a reduction in the teeth’s working surface to a degree that makes the further transmission of loads (in the case of gears) impossible. In some situations, the teeth of carbonized wheels can change their shape to such a degree that it leads to their significant wear or even fracture as a result of pitting. The carbonized and hardened teeth of wheels, which were subjected to pitting in the area of the tooth root connected to significant wear, were vulnerable to fracture. The reason for this was the initiation and development of fatigue cracks in the pits generated where the material that crumbled away was. A significant noise accompanies the operation of gear wheels with considerable pitting [27].

**Figure 16:**
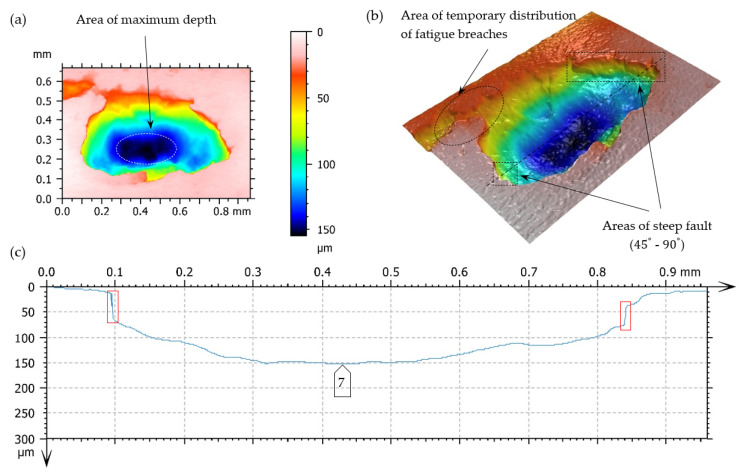
Fatigue pit (pitting) on the tooth’s working surface—magnitude 10×. (**a**,**b**) Superficial 3D view—pseudocolour of surface with visualization of depth, (**c**) profile curve, -----—cross-section line, □—indication of breach edges, inclined at an angle close to 90° to the surfaces under analysis, on the cross-section.

**Figure 17:**
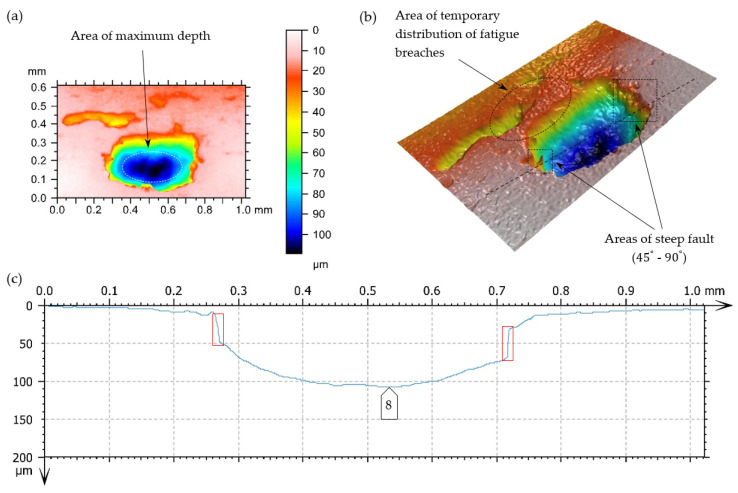
Fatigue pit (pitting) on the tooth working surface—magnitude 10×. (**a**,**b**) Superficial view 3D—pseudocolour of surface with visualization of depth, (**c**) profile curve, -----—line of cross-section, □—indication of breach edges, inclined at an angle close to 90° to the surfaces under analysis, on the cross-section.

A surface’s increase in pitting cavities is caused very often by their mutual connection. In Figure 16 and Figure 17, the limiting state before the connection of two fatigue breaches is presented.

The main reason for generating this type of breach includes exceeding the limit of contact stresses of the teeth meshing surfaces. The degree of tolerating these kinds of defects (their number, size, and arrangements) normally depends mainly on the application area. In this case, (based on the assumption of the forecasted pump life due to the degradation development of the top layer of meshing teeth), it does not have any big significance. Pitting breaches in the scope of the dimensions 0.1–1.5 mm (Figure 14) and even 2.5 mm (Figure 15) do not exclude the pinion from its further (but temporary) exploitation. It should be borne in mind that correct filtration should be ensured. In this case, a high-pressure filter of exact purification should be installed directly after the pump to prevent the failure of valves and receivers situated in the hydraulic line between the pump and the tank, caused by solid impurities.

Additionally, it should be taken into account that a breach of 1 mm dia. close to the fillet of the tooth heat-treated or superficially hardened may begin a crack, which can finally lead to a tooth break; this is why, in some cases, this kind of breach should be treated as inadmissible (e.g., in air applications) [50].

**Figure 18:**
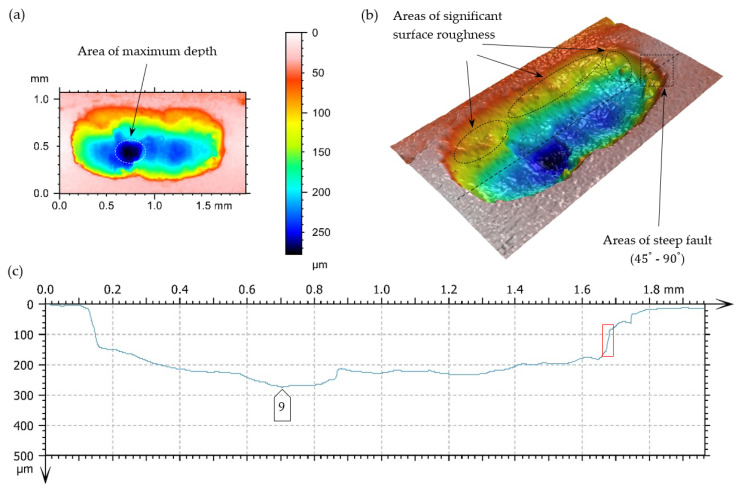
Fatigue pit (pitting) on the tooth’s working surface—magnitude 10×. (**a**,**b**) Superficial 3D view—pseudocolour of surface with visualization of depth, (**c**) profile curve, -----—line of cross-section, □—indication of breach edges, inclined at an angle close to 90° to the surfaces under analysis, on the cross-section.

Destructive pitting develops naturally from non-destructive pitting, micropitting, and that one in turn from a dull finish of the surface and imperfections in the top layer of meshing teeth. An essential issue also concerns characteristics of chipping (Figure 14, Figure 15, Figure 16, Figure 17 and Figure 18), as well as the relationship between its depth and the thickness of the top layer, subjected to heat treatment or thermochemical treatment. The present measurement technologies (optical metrology) enable us to check the real depth of this type of defect.

To determine the essence of top layer wear of the pinion selected working surfaces under testing, a short geometric analysis of individual chippings was performed. In Table 4, basic measurement values, characterizing the geometries of defects, are presented, i.e., the maximum depths of pitting chippings are compared to the changes in the macrostructure resulting from the applied thermochemical treatment.

**Figure 19:**
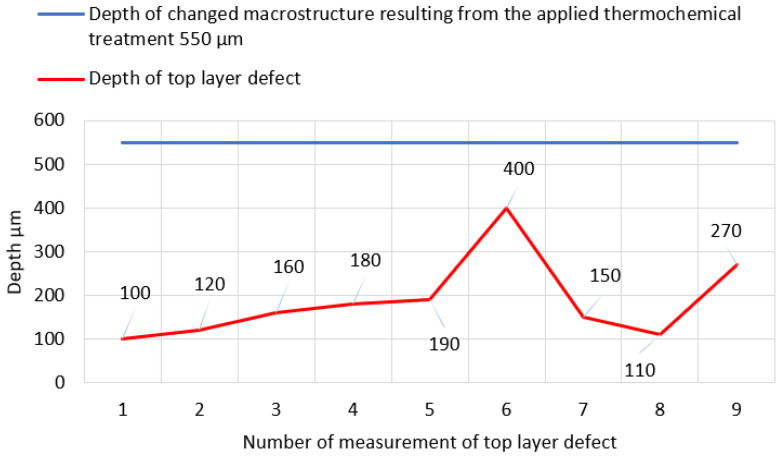
Graphical presentation of the data from Table 4.

In the cases under analysis, the defect depth of the top layer did not exceed the depth of the layer subjected to thermochemical treatment. The location of all chippings is enclosed in the scope of the zone, with the structure of a low-temperature tempered, fine acicular martensite of comparatively decreasing carbon contents. The measurement data from Table 4 are graphically presented in Figure 19.

**Figure 20:**
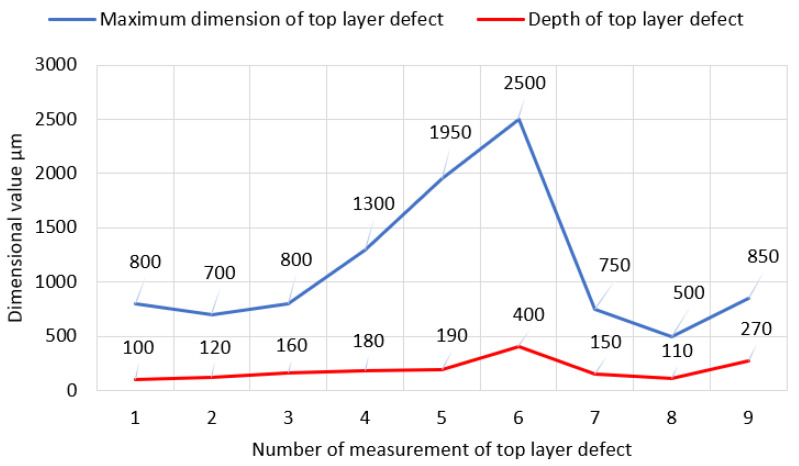
Graphical presentation of the data from Table 5.

In most cases, the correlation between the maximum dimension of the top-layer defect in relation to the adequate deeper chipping was confirmed—Figure 20.

## 7. Conclusions

The concept of the paper was to carry out an extensive microscopic and macroscopic analysis of the degree of wear of the surface layer of a thermo-chemically treated pinion. Despite the realization of the assumptions made, the topic is still open. The complex tribological phenomena occurring in the interface of such important machine parts as cogwheels still offer many opportunities for scientific research. The prediction of the fatigue strength of the diffusion layer and the selection of optimum parameters for thermal/chemical treatment processes must be supported by appropriate diagnostic methods. We are convinced that optical metrological technology will continue to develop and offer opportunities for even greater measurement precision.

Based on the results of our study, it was found that the thermo-chemical treatment carried out only partially met the design objectives (in the sense of achieving the corresponding macrostructure changes within the material). In all cases tested, the depth of the micropits did not exceed the depth of the diffusion layer. The results obtained from the evaluation of the 3D scans allow, to a certain extent, a qualitative verification of the applied thermo-chemical treatment. The determinants of the evaluation were the following parameters: defect profile, depth, bottom roughness, and nature of the recess (steep or mild).

In addition, the high content (up to 30%) of residual austenite in the volume of hardened surfaces enables us to formulate a hypothetical conclusion concerning the non-optimum parameters of a thermochemical process in relation to the chemical composition of the pinion under analysis.

The occurrence of residual austenite causes a reduction in tooth surface hardness, the initiation of brittle fractures, a rapid reduction in fatigue strength and an increase in brittleness; in particular, it can indicate a trend of generating fractures in the superficial layer during an exploitation.

Additionally, as a result of the conducted analysis of chemical composition with the use of the spectral method, it can be stated that in the scope of the elements under analysis, the material of the specimen, subjected to tests, does not have its direct equivalent in the group of alloy machine steels used for carbonization according to the standard PN-EN ISO 683:2022-07. The application of steel grades, determined by the standards in effect, enables one to avoid the presence of other elements, which can affect the properties of the material. The maintenance of the correct chemical composition will ensure the required strength, hardness and other mechanical parameters, which play a key role in ensuring the correct properties.

Moreover, to extend the life of gear wheels and pinions, their heat treatment should be selected properly. In the case of heavily loaded gear wheels, it is recommended to use sub-zero treatment, enabling the very efficient minimization of residual austenite contents and thus an improvement in hardness and dimensional stabilization.

Additional information concerning the recommendations and advantages resulting from heat treatment is as follows:

- The microstructure of the specimen, after the present heat treatment, subjected to tests, indicates a partial achievement of the project’s assumptions, but with the presence of residual austenite;
- The high content of residual austenite in the hardened surface volume suggests the non-optimum parameters of the heat treatment process in relation to the chemical composition of pinion subjected to tests.

Advantages resulting from heat treatment:

- A life extension of gear wheel parts through the minimization of residual austenite contents;
- An improvement in tooth surface hardness and dimensional stabilization;
- A reduction in initiations of brittle fractures, an improvement in fatigue strength, and a reduction in brittleness;
- Protection against the generation of fractures in the top layer during an exploitation.

In relation to the information given above, it is recommended to apply additional heat treatments in the form of sub-zero treatments, which will enable the efficient minimization of the residual austenite contents, an improvement in hardness, and dimensional stabilization which will contribute towards the extension of the pinion’s life and an improvement in its useful properties.

We believe that the presented topic provides a reasonable basis for further research: (a) the adequate measurement analysis of an identical cogwheel subjected to low-temperature treatment and (b) optical measurements of surface defects occurring below the diffusion layer. It is hoped that the implementation of the planned activities will generate a discussion and allow answers to several hypothetical questions to be formulated.

## Figures and Tables

**Table 1:** Determination of the conventional depth of hardening after carburizing by measuring the hardness distribution deep into the material—A side.

Distance from surface, mm	0.04	0.09	0.14	0.19	0.24	0.29	0.34	0.39	0.44	0.49	0.54
HV hardness	727.2	728.1	729.4	721.9	702.1	692.9	671.3	640.3	622.9	582.1	579.6
Distance from surface, mm	0.59	0.64	0.69	0.74	0.79	0.84	0.89	0.94	1.44	1.94	
HV hardness	548.9	527.6	525.5	508.8	488.2	486.9	482.4	471.7	455.4	442.5	

**Table 2:** Determination of the conventional depth of hardening after carburizing by measuring the hardness distribution deep into the material—B side.

Distance from surface, mm	0.04	0.09	0.14	0.19	0.24	0.29	0.34	0.39	0.44	0.49	0.54
HV hardness	695.9	718.2	722.1	691.3	686.0	667.9	657.9	630.7	610.5	590.4	568.9
Distance from surface, mm	0.59	0.64	0.69	0.74	0.79	0.84	0.89	0.94	1.44	1.94	
HV hardness	534.7	525.5	523.1	509.4	474.4	485.5	482.4	475.2	436.8	447.9	

**Table 3:** Average value (from three measurements) of the specimen’s chemical composition [27], and the requirements of the standard PN-EN 10084:2002.

**%**	**C**	**Mn**	**Si**	**P**	**S**	**Cr**	**Ni**	**Mo**	**V**
Tested material	0.2140	0.6350	0.3510	0.0160	0.0030	1.3300	1.6400	0.2670	0.0060
18CrNiMo7-6 acc. to PN-EN 10084:2002	0.14–0.19	0.40–0.70	0.17–0.37	0.035	0.035	1.50–1.80	1.40–1.70	0.25–0.35	–
**%**	**Cu**	**Al**	**Ti**	**Nb**	**Co**	**As**	**B**	**Pb**	**Zr**
Tested material	0.1790	0.0180	0.0030	0.0000	0.0140	0.0210	0.0020	0.0090	0.0030
18CrNiMo7-6 acc. to PN-EN 10084:2002	–	–	–	–	–	–	–	–	–

**Table 4:** Geometric characteristics of chippings and an analysis of selected values in relation to the depth changes in macrostructure resulting from the applied superficial thermochemical treatment.

I	II µm	III µm
1	550	100
2	550	120
3	550	160
4	550	180
5	550	190
6	550	400
7	550	150
8	550	110
9	550	270

I—No. of defect, II—depth of changed macrostructure resulting from the applied thermochemical treatment, III—maximum depth of chipping measured in relation to the local flank pitch line surface in the defect vicinity (±5 μm).

**Table 5:** Relationship between maximum dimension of analyzed chippings and their corresponding depth values.

I	II mm	III µm
1	0.8	100
2	0.7	120
3	0.8	160
4	1.3	180
5	1.95	190
6	2.5	400
7	0.75	150
8	0.5	110
9	0.85	270

I—No. of defect, II—maximum dimension of chipping measured in parallel to the tooth flank pitch line (±0.05 mm), III—maximum depth of chipping measured in relation to the local flank pitch line surface in the defect vicinity (±5 μm).

## Data Availability

The original contributions presented in the study are included in the article, further inquiries can be directed to the corresponding author/s.
